# Intraocular pressure according to different types of tonometry (non-contact and Goldmann applanation) in patients with different degrees of bilateral tearing

**DOI:** 10.1371/journal.pone.0222652

**Published:** 2019-09-16

**Authors:** Bo Ram Seol, Tae Gu Kang, Bonhyeok Gu

**Affiliations:** Department of Ophthalmology, Veterans Health Service (VHS) Medical Center, Seoul, Korea; Keio University School of Medicine, JAPAN

## Abstract

**Purpose:**

To investigate intraocular pressure (IOP) readings by non-contact tonometry (NCT) and Goldmann applanation tonometry (GAT) for patients with different degrees of bilateral tearing.

**Methods:**

In this study, we reviewed the medical charts of patients complaining of different degrees of bilateral tearing. The tear meniscus height (TMH) and IOP with NCT and GAT were measured. In each patient, a comparison of IOP readings between the eye with lower TMH and the contralateral eye with higher TMH was evaluated. The TMH was graded as follows: grade 1 (low): TMH < 0.2 mm; grade 2 (moderate): 0.2 mm ≤ TMH < 0.6 mm; grade 3 (high): TMH ≥ 0.6 mm. Subsequently, a comparison of IOP readings among eyes with low, moderate, and high TMH was also performed.

**Results:**

A total of 120 eyes of 60 patients were enrolled. When comparing the two eyes of a patient, the eye with higher TMH showed higher NCT readings and larger difference in IOP readings between the two tonometries than the eye with lower TMH (P < 0.001 and P < 0.001, respectively). When TMH was classified into grades according to the degree, the high TMH eyes showed higher NCT readings than did the low and moderate TMH eyes (P < 0.001 and P = 0.001, respectively). In addition, the high TMH eyes showed a larger difference in IOP readings between the two tonometries than did the low and moderate TMH eyes (P < 0.001 and P < 0.001, respectively).

**Conclusion:**

Eyes with higher TMH showed higher NCT readings and a larger difference in IOP between the two tonometries (NCT and GAT) than those with lower TMH. In patients with tearing, the NCT value may be inaccurate, so it is necessary to measure the GAT.

## Introduction

Accurate measurement of intraocular pressure (IOP) is a very important part of ophthalmic examinations. There are many ways to measure IOP. Goldmann applanation tonometry (GAT) is the gold standard of IOP measurement because of its known accuracy. Non-contact tonometry (NCT) is one of the most commonly used modalities in primary-care clinical practice, in which setting it has many advantages over GAT [1–4] NCT is easy to use and provides an objective means of IOP measurement that paramedical personnel with minimal training can perform as effectively as physicians [5, 6]. Additionally, unlike other instruments, NCT do not require local anesthetics or fluoroscein. Its use also avoids the potential complications of corneal abrasions, infection, and drug sensitivities, because it is not in direct contact with the eye. Thus, it can be used in patients who often are uncooperative (most commonly the elderly and children) [1–4]

Also, NCT IOP readings show less variability than GAT ones [7]. There are several factors that affect IOP readings. Especially, corneal factors such as thickness, curvature, and elasticity are known to be related to IOP measurement [8–13]. Among the several factors that are known to affect IOP readings, little is known about the effect of tearing, even though patients complaining of tearing are often seen in clinical practice. About one-third of outpatients visiting ophthalmic clinics have tearing or complications associated with it [14]. The degree of such tearing can be roughly determined by listening to patients’ reported symptoms or, more precisely, by measuring the tear meniscus height (TMH) under slit lamp. With GAT, the thicker the ring, the higher the IOP that is measured [15].The effect of tearing on NCT, however, is not yet clear.

To the best of our knowledge, there has been no study on the effect of tearing on IOP readings, especially NCT ones. Thus, this study was conducted to evaluate NCT and GAT readings and binocular differences of IOP in patients with different degrees of bilateral tearing.

## Materials and methods

### Subjects

We reviewed the medical records of patients complaining of different degrees of bilateral tearing during the period from March 2017 to August 2017 at VHS Medical Center. The study followed the tenets of the Declaration of Helsinki, and was approved by the Institutional Review Board of VHS Medical Center. Due to the retrospective nature of the data analysis, the Institutional Review Board of VHS Medical Center determined that informed consent as signed by patients was not necessary. Patients’ information was anonymized and de-identified prior to the analysis.

All of the subjects underwent an ophthalmic examination that included visual acuity (VA), IOP measurement, corneal pachymetry (Tomey SP-3000 Pachymeter; Tomey Corp, Nagoya, Japan), and lacrimal irrigation.

Patients were excluded if they showed regurgitation due to lacrimal drainage system obstruction in a lacrimal syringing test. Patients with a history of lacrimal drainage system surgery such as silicone tube intubation or dacryocystorhinostomy also were excluded. In addition, the patients with corneal disease were also excluded because it may affect the IOP readings.

The TMH was measured at the primary position using a 1- or 0.2 mm slit beam by slit lamp microscopy. It was measured from the cornea-meniscus junction to the lower lid-meniscus junction with a scan line perpendicular to the mucocutaneous junction [16]. A previous study reported that the median TMH in eyes with nasolacrimal obstruction was 0.6 mm and in control eyes, 0.2 mm [17]. Thus, we defined TMH < 0.2 mm as grade 1: low, 0.2 mm ≤ TMH ≤ 0.6 mm as grade 2: moderate; and TMH > 0.6 mm as grade 3: high. All of the measurements were performed by one glaucoma specialist (B.R.S.) prior to irrigation.

In all of the patients, IOP was measured by both NCT and GAT. The NCT reading was obtained with one device (Topcon CT80 Computerized Tonometer; Topcon, Tokyo, Japan). The average of three consecutive measurements obtained by an NCT (CT80; Topcon Co., Tokyo, Japan) was regarded as the NCT result. The IOP assessment with the GAT was always subsequent to that with the non-contact tonometer. The GAT reading was obtained with one calibrated device (Haag-Streit AG Goldmann Tonometer). Following topical corneal anesthesia (Proparacaine Hydrochloride 0.5%, Alcon) and fluorescein staining, a series of three successive measurements was obtained, the instrument being reset to the zero position between readings. The IOP measurements with GAT were performed by the one glaucoma specialist (B.R.S.). The difference in IOP readings between the two tonometries was defined as follows: IOP with NCT–IOP with GAT. After the NCT measurement, the TMH was measured, followed by the GAT measurement.

### Statistical analysis

Comparisons of the grades of TMH, NCT and GAT readings, differences in IOP readings between the two tonometries, and central corneal thickness (CCT) between eyes with lower TMH eyes and contralateral higher TMH eyes were performed by paired t-test. The NCT and GAT readings as well as the differences between them were compared among the 3 groups divided by the degree of TMH using generalized least squares regression. Comparison of IOP readings in patients with small binocular TMH difference and those with large binocular TMH difference were performed by Mann-Whitney test. Comparison of IOP between NCT readings and GAT readings were performed by Wilcoxon signed rank test. All of the statistical analyses were performed using SPSS 18.0 (SPSS, Inc. Chicago, IL, USG) and R 3.5.1 (R Foundation, Vienna, Austria); P values less than 0.05 were considered statistically significant.

## Results

### Subjects

The study initially included 196 eyes of 98 patients complaining of different degrees of bilateral tearing. Of these, 28 patients were excluded because they did not show different grades of TMH between their eyes. Another 10 patients were excluded because they did not have IOP measurement readings from both NCT and GAT. Finally, 120 eyes (45 eyes with low TMH, 48 eyes with moderate TMH, and 12 eyes with high TMH) of 60 patients were included in this study. The demographics and baseline characteristics of the subjects are summarized in Table 1. The mean age of the subjects was 74.56 ± 4.06 years, and the mean CCT was 554.78 ± 24.98 μm. The mean grade of TMH and the mean NCT and GAT readings were 1.85 ± 0.75, 15.04 ± 2.90 mmHg, and 13.84 ± 2.49 mmHg, respectively.

**Table 1:** Clinical characteristics of patients included in the study.

Characteristic	Total patients (n = 60) Mean ± SD or n (%)			
Age (years)	74.56 ± 4.06			
Sex				
Male	47 (78.3)			
Female	13 (21.7)			
Diabetes	20 (33.3)			
Systemic hypertension	36 (60.0)			
	Total eyes (n = 120) Mean ± SD or n (%)	Eyes with low TMH grade (n = 45)	Eyes with moderate TMH grade (n = 48)	Eyes with high TMH grade (n = 27)
Grade of TMH	1.85 ± 0.75	1.00 ± 0.00	2.00 ± 0.00	3.00 ± 0.00
NCT readings (mmHg)	15.04 ± 2.90	14.44 ± 2.76	14.96 ± 2.97	16.19 ± 2.76
GAT readings (mmHg)	13.84 ± 2.49	13.80 ± 2.28	14.12 ± 2.76	13.41 ± 2.34
NCT-GAT readings (mmHg)	1.20 ± 2.52	0.64 ± 2.30	0.83 ± 2.52	2.78 ± 2.31
CCT (μm)	554.78 ± 24.98	554.10 ± 22.52	552.7 ± 43.13	560.30 ± 21.21
Other combined disease				
Cataract	39 (32.5)	17 (37.8)	16 (33.3)	6 (22.2)
Retinal disease	9 (7.5)	5 (11.1)	2 (4.2)	2 (7.4)
Glaucoma	35 (29.2)	13 (28.9)	11 (22.9)	11 (40.7)

SD, standard deviation; TMH, tear meniscus height; NCT, non-contact tonometer; GAT, Goldmann applanation tonometer; CCT, central corneal thickness

### Grade of TMH, IOP measured with NCT and GAT

As shown in Table 2, there was a statistically significant difference in the grade of TMH between eyes with higher TMH and those with lower TMH (P < 0.001). There was no significant difference in CCT between the eyes with higher TMH and those with lower TMH eyes (P = 0.519). The higher TMH eyes showed higher NCT readings and a larger difference in IOP readings between the two tonometries than did the lower TMH eyes (P < 0.001 and P < 0.001, respectively).

**Table 2:** Grade of tear meniscus height, intraocular pressure measured with non-contact tonometer and Goldmann applanation tonometer.

	Eyes with lower TMH	Eyes with higher TMH	Difference (higher THM eyes-lower TMH eyes)	P-value
	(n = 60) mean ± SD or n (%)	(n = 60) mean ± SD or n (%)		
Grade of TMH	1.25 ± 0.44	2.45 ± 0.50		**< 0.001**
Grade of TMH				
low	45 (75.0)	0 (0.0)		
moderate	15 (25.0)	33 (55.0)		
high	0 (0.0)	27 (45.0)		
NCT readings, mmHg	14.28 ± 2.75	15.80 ± 2.87	1.52 ± 2.76	**< 0.001**
GAT readings, mmHg	13.67 ± 2.30	14.02 ± 2.68	0.35 ± 1.96	0.173
Difference in IOP readings between the two tonometries, mmHg	0.62 ± 2.27	1.78 ± 2.64	1.17 ± 2.44	**< 0.001**
CCT, μm	555.56 ± 22.70	554.00 ± 27.52	-1.56 ± 11.92	0.519

TMH, tear meniscus height; SD, standard deviation; IOP, intraocular pressure; NCT, non-contact tonometer; GAT, Goldmann applanation tonometer; CCT, central corneal thickness. Difference in IOP readings between two tonometries was defined as NCT reading–GAT reading. Paired t-test; bolded values represent significance, P < 0.05

### Comparison of IOP readings among eyes with low, moderate, and high TMH

There was no significant difference in GAT readings among eyes with low, moderate, and high TMH grades (P = 0.245). There were significant differences in NCT and in IOP readings between the two tonometries among the low, moderate, and high TMH eyes (P < 0.001 and P < 0.001, respectively). The high TMH eyes showed higher NCT readings than did the low and moderate TMH eyes (P < 0.001 and P = 0.001, respectively). The high TMH eyes also showed larger difference in IOP readings between two tonometries than did the moderate or low TMH eyes (P < 0.001 and P < 0.001, respectively) (Table 3).

**Table 3:** Comparison of intraocular pressure readings between eyes with low, moderate, and high tear meniscus height.

	Eyes with low TMH grade (n = 45)	Eyes with moderate TMH grade (n = 48)	Eyes with high TMH grade (n = 27)	Comparison	Difference (95% CI)	P-value
	mean ± SD, median [IQR]	mean ± SD, median [IQR]	mean ± SD, median [IQR]			
NCT readings, mmHg	14.44 ± 2.76, 15.00 [13.00;16.00]	14.96 ± 2.97, 15.00 [13.00;17.00]	16.19 ± 2.76, 16.00 [15.00;17.50]			**< 0.001**^**a**^
				Grade 1 vs 2	0.57 (-0.40~1.53)	0.351^b^
				Grade 2 vs 3	1.84 (0.62~3.06)	**0.001**^**b**^
				Grade 1 vs 3	2.41 (1.16~3.65)	**< 0.001**^**b**^
GAT readings, mmHg	13.80 ± 2.28, 14.00 [12.00;15.00]	14.12 ± 2.76, 13.00 [12.00;16.00]	13.41 ± 2.34, 13.00 [12.00;15.00]			0.245^a^
Difference in IOP readings between the two tonometries, mmHg	0.64 ± 2.30, 1.00 [-1.00;2.00]	0.83 ± 2.52, 0.50 [-1.00;2.00]	2.78 ± 2.31, 3.00 [1.50;4.50]			**< 0.001**^**a**^
				Grade 1 vs 2	0.08 (-0.71~0.86)	0.972^b^
				Grade 2 vs 3	2.29 (1.27~3.30)	**< 0.001**^**b**^
				Grade 1 vs 3	2.36 (1.33~3.39)	**< 0.001**^**b**^

TMH, tear meniscus height; SD, standard deviation; IQR, interquartile range; CI: confidence interval; IOP, intraocular pressure; NCT, non-contact tonometer; GAT, Goldmann applanation tonometer; CCT, central corneal thickness. Difference in IOP readings between two tonometries was defined as NCT reading–GAT reading

^a^P-value from generalized least squares regression models adjusted age, sex, systemic hypertension, diabetes; bolded values represent significance, P < 0.05

^b^P-value for pairwise comparisons adjusted using the Tukey method; bolded values represent significance, P < 0.05

### Comparison of IOP readings in patients with small binocular TMH difference and large binocular TMH difference

There was no significant binocular difference in NCT and GAT readings, and difference in IOP readings in IOP readings between the two tonometries comparing patients with small binocular TMH difference and those with large binocular TMH difference (P = 0.234, 0.925, and 0.052, respectively) (Table 4).

**Table 4:** Comparison of intraocular pressure readings in patients with small and large binocular tear meniscus height difference.

		Patients with small binocular TMH difference	Patients with large binocular TMH difference	P-value
		(n = 48)	(n = 12)	
Binocular difference in NCT readings, mmHg	mean ± SD	1.25 ± 2.66	2.58 ± 3.00	
	median [IQR]	1.00 [-1.00;3.50]	2.50 [1.50;4.00]	0.234
Binocular difference in GAT readings, mmHg	mean ± SD	0.35 ± 1.94	0.33 ± 2.15	
	median [IQR]	0.00 [-0.50;1.00]	0.50 [-1.00;2.00]	0.925
Binocular difference of difference in IOP readings between the two tonometries, mmHg	mean ± SD	0.90 ± 2.45	2.25 ± 2.14	
	median [IQR]	1.00 [-1.00;3.00]	3.00 [1.00;4.00]	0.052

TMH = tear meniscus height; SD = standard deviation; IQR, interquartile range; IOP = intraocular pressure; NCT = non-contact tonometer; GAT = Goldmann applanation tonometer. Binocular difference in grade of TMH was defined as TMH grade of the eye with higher TMH–the TMH grade of the eye with lower TMH. Difference in IOP readings between the two tonometers was defined as NCT reading–GAT reading. Mann-Whitney test; bolded values represent significance, P < 0.05

### Comparison of IOP between the NCT readings and GAT readings

The GAT readings were lower than the NCT readings in both eyes, i.e. lower and higher TMH eyes (P = 0.040 and P < 0.001, respectively). In addition, the GAT readings were lower than the NCT readings in eyes with moderate and high TMH grade (P = 0.040 and P < 0.001, respectively). The binocular difference in GAT readings were smaller than those in NCT readings in both small and large binocular TMH difference patients (P = 0.013 and P = 0.012, respectively) (Table 5).

**Table 5:** Comparison of intraocular pressure between the non-contact tonometry and Goldmann applanation tonometry readings.

	NCT readings (mmHg)	GAT readings (mmHg)	Difference (NCT- GAT reading)	P-value
Eyes with lower TMH (n = 60)				
mean ± SD	14.28 ± 2.75	13.67 ± 2.30	0.62 ± 2.27	
median [IQR]	14.50 [13.00;16.00]	13.00 [12.00;15.50]		**0.040**
Eyes with higher TMH (n = 60)				
mean ± SD	15.80 ± 2.87	14.02 ± 2.68	1.78 ± 2.64	
median [IQR]	16.00 [14.00;17.00]	13.50 [12.00;16.00]		**< 0.001**
Eyes with low TMH grade (n = 45)				
mean ± SD	14.44 ± 2.76	13.80 ± 2.28	0.64 ± 2.30	
median [IQR]	15.00 [13.00;16.00]	14.00 [12.00;15.00]		0.053
Eyes with moderate TMH grade (n = 48)				
mean ± SD	14.96 ± 2.97	14.12 ± 2.76	0.83 ± 2.52	
median [IQR]	15.00 [13.00;17.00]	13.00 [12.00;16.00]		**0.040**
Eyes with high TMH grade (n = 27)				
mean ± SD	16.19 ± 2.76	13.41 ± 2.34	2.78 ± 2.31	
median [IQR]	16.00 [15.00;17.50]	13.00 [12.00;15.00]		**< 0.001**
	Binocular difference in NCT readings (mmHg)	Binocular difference in GAT readings (mmHg)	Difference (NCT–GAT reading)	P-value
Patients with small binocular TMH difference (n = 48)				
mean ± SD	1.25 ± 2.66	0.35 ± 1.94	0.90 ± 2.45	
median [IQR]	1.00 [-1.00;3.50]	0.00 [-0.50;1.00]		**0.013**
Patients with large binocular TMH difference (n = 12)				
mean ± SD	2.58 ± 3.00	0.33 ± 2.15	2.25 ± 2.14	
median [IQR]	2.00 [1.50;4.00]	0.50 [-1.00;2.00]		**0.012**

TMH = tear meniscus height; NCT = non-contact tonometer; GAT = Goldmann applanation tonometer; CCT = central corneal thickness; SD = standard deviation; IQR = interquartile range. Binocular TMH difference was defined as, TMH grade of the eye with higher TMH–the TMH grade of the eye with lower TMH. Wilcoxon signed rank test; bolded values represent significance, P < 0.05

## Discussion

Previously, a few studies have reported that after instillation of artificial tears, IOP measured with NCT increased. Ehlers et al. studied the effects of artificial tears on NCT measurements. They measured IOP using the American Optical Non-contact Tonometer (AONGT) after one drop and also after two drops of artificial tears. In the results, the mean IOP was elevated by 1.5 mmHg when using one eye drop and by 1.8 mmHg when using two eye drops [9]. Similarly, KC Lam A et al. measured IOP with the Pulsair tonometer after instillation of artificial tears, and they also showed an increase in IOP, though it was not statistically significant. They noted that when measuring IOP with NCT, it may be necessary to take several measurements, because patients reflexively shed tears after the first one. In fact, given that refluxing tears can occur due to the instillation of artificial tears, the TMH at the time of IOP measurement might vary per measurement, because the volume of such tears varies. To overcome this limitation, it may be necessary to confirm the TMH at the time of IOP measurement. Thus, in the present study, we did not use artificial tears; rather, we focused on the IOP difference between eyes with different bilateral TMH.

In this study, we performed various methods to determine the effect of TMH on IOP readings. First, the relatively low TMH and contralateral high TMH eyes were compared in each patient. Second, all 120 eyes were divided into 3 groups according to the TMH grade and analyzed. In this analysis, low, moderate, and high mean TMH grades which are classified according to TMH values. Our results showed that eyes with higher TMH showed higher NCT readings and a larger difference in IOP readings between the two tonometries (NCT reading–GAT reading) than did contralateral lower TMH eyes. Also, the mean difference was a positive value for both eyes with higher TMH and those with lower TMH, indicating that the NCT readings were higher than the GAT readings, and also higher in the eyes with higher TMH than in those with lower TMH. Similar results were obtained when the 120 eyes were graded according to the degree of TMH and divided into 3 groups. In addition, as shown in Table 5, there was also a significant difference in comparing NCT and GAT in each group, which is consistent with the previous analysis.

In particular, it is well known that IOP is measured more highly when the ring width is thick in IOP measurement performed with GAT [4]. If the TMH is high, the ring width is also thick, in which cases the TMH can affect the value of IOP measured with GAT. However, when we measured IOP with GAT in this study, the effect of tearing might have been slight, because one ophthalmologist (B.R.S.) wiped out tears and measured the IOP in the state of a constant ring width for all patients. The fluorescein semicircle width was approximately 10% of the diameter of the applanation surface. With this method, we tried to eliminate the effect of tearing on GAT readings. Therefore, it can be assumed that the effect of tearing on the GAT reading was removed and that only the effect of tearing on the NCT reading could have affected the results of this study. Indeed, there were no significant differences in GAT readings among eyes with low, moderate, and high TMH grade.

Our study has several limitations. First, it included only a relatively small number of patients. We could not enroll a large number of subjects, because we included patients who had at least a 1 grade of binocular TMH difference. Thus, there is a need to carry out the study with a larger number of subjects in the future. Second, IOP measured by NCT might be somewhat different from that measured by GAT, even if the effects of tearing are excluded. In the normal range of IOP, the agreement between the two tonometries is known to be as high as approximately 99.0%; however, for the higher IOP ranges, NCT shows decreased reliability [18, 19]. To overcome this limitation, we enrolled only patients showing a normal range of IOP. Third, it is not yet clear how much difference in IOP is clinically meaningful. Especially, it may not meaningful that the difference in IOP between 1 and 2 in the normal range of IOP. However, for example, in patients with glaucoma, the difference from 1 to 2 of IOP may account for 10–20% of baseline IOP. Thus, it may be meaningful in some patients.

In conclusion, we found that the NCT readings are high and the difference in IOP measured with the two different tonometries (NCT and GAT) is large when the TMH is high. This study suggests that we should be aware of the fact that the IOP, especially measured with NCT may be inaccurate especially in patients with tearing. Thus, it is necessary to measure GAT for these patients. In addition, if the tearing in each eye is different, the IOP readings between the eyes may be different.

## Supporting information

S1 Table Grade of TMH, IOP measured with NCT and GAT. (XLSX)
